# Genome Sequences of *Vibrio navarrensis*, a Potential Human Pathogen

**DOI:** 10.1128/genomeA.01188-14

**Published:** 2014-11-20

**Authors:** Lori M. Gladney, Lee S. Katz, Kristen M. Knipe, Lori A. Rowe, Andrew B. Conley, Lavanya Rishishwar, Leonardo Mariño-Ramírez, I. King Jordan, Cheryl L. Tarr

**Affiliations:** a National Center for Emerging Zoonotic and Infectious Diseases, Centers for Disease Control and Prevention, Atlanta, Georgia, USA; b IHRC, Inc., Atlanta, Georgia, USA; c School of Biology, Georgia Institute of Technology, Atlanta, Georgia, USA; d National Center for Biotechnology Information, National Library of Medicine, National Institutes of Health, Bethesda, Maryland, USA

## Abstract

*Vibrio navarrensis* is an aquatic bacterium recently shown to be associated with human illness. We report the first genome sequences of three *V. navarrensis* strains obtained from clinical and environmental sources. Preliminary analyses of the sequences reveal that *V. navarrensis* contains genes commonly associated with virulence in other human pathogens.

## GENOME ANNOUNCEMENT

*Vibrio navarrensis* is a Gram-negative bacterium that was first described from sewage in the province of Navarra, Spain in 1982 (1). The bacterium has received scant attention since its description and so little is known about its ecology. Recently Gladney and Tarr (2) characterized *V. navarrensis* isolates that were recovered from various clinical sources, including blood, suggesting its potential to be a human pathogen. *V. navarrensis* is genetically and phenotypically similar to *Vibrio vulnificus* (2, 3), an opportunistic pathogen with a high case fatality rate (>50% mortality rate in septicemic individuals) (4). We determined the genome sequence for three isolates of *V. navarrensis* to gain insight into its metabolic functions, including potential virulence mechanisms, and to search for possible diagnostic markers to distinguish it from *V. vulnificus*.

The whole-genome sequence of type strain ATCC 51183 (also LMG 15976 and 1397-6T, from sewage) was determined on a Pacific Biosciences *RS* instrument using P4-C2 chemistry and four single-molecule real-time (SMRT) cells (Pacific Biosciences, Menlo Park, CA). The Nextera XT sample preparation kit (Illumina, San Diego, CA) was used to generate libraries for isolates 0053-83 (human wound) and 08-2462 (human blood) and 150-bp paired-end reads were generated on a MiSeq (Illumina). The genomes were sequenced to a depth of 70 to 215× (average, 128×).

*De novo* assembly of the sequence for type strain ATCC 51183 was performed using the Hierarchical Genome Assembly Process (HGAP) in SMRT Analysis version 2.0 (available at http://www.pacb.com/devnet/) (5), which resulted in a closed genome with two contigs representing the two chromosomes. *De novo* assembly of 0053-83 and 08-2462 was performed using CG-Pipeline version 0.4.1, which leverages VelvetOptimiser and Velvet (CGP available at http://sourceforge.net/projects/cg-pipeline) (6, 7), resulting in 64 and 115 contigs, respectively. The GC content is 49% for all three genomes, and assembly lengths range from 4.2 to 4.4 Mb (average, 4.3 Mb). The genomes were annotated with the NCBI Prokaryotic Genomes Automatic Annotation Pipeline (PGAAP) and Rapid Annotation using Subsystem Technology (RAST) (8, 9). The number of predicted genes range from 3,740 to 3,982 (average, 3,829), including 38 to 94 pseudogenes (average, 58) and 78 to 138 RNAs (average, 100).

A number of genes that are associated with virulence in other human pathogens were found in the *V. navarrensis* genome sequence. RAST annotated 492 subsystems, which included sequences for integrases, iron-acquisition, capsular polysaccharide biosynthesis, bacteriocins, metal resistance, multidrug resistance efflux pumps, and four secretion systems (types I, II, IV, and VI). Of importance, the type IV secretion system has been shown to be associated with natural competence, conjugation, and translocation of effector proteins and toxins, while the type VI secretion system appears to mediate competition between heterologous bacterial species, including bactericidal activity (10, 11). Taken together, the gene content and the recovery from normally sterile sites in humans suggest that *V. navarrensis* may be a previously unrecognized human pathogen with capabilities to cause systemic disease, perhaps opportunistically, like its close relative *V. vulnificus*.

### Nucleotide sequence accession numbers.

This whole-genome shotgun project has been deposited at DDBJ/EMBL/GenBank under the accession numbers JMCF00000000, JMCG00000000, and JMCI00000000. The versions described here are the first versions: JMCF01000000, JMCG01000000, and JMCI01000000.
